# At the Nexus of History, Ecology, and Hydrobiogeochemistry: Improved Predictions across Scales through Integration

**DOI:** 10.1128/mSystems.00167-17

**Published:** 2018-04-10

**Authors:** James C. Stegen

**Affiliations:** a Pacific Northwest National Laboratory, Earth and Biological Sciences Directorate, Richland, Washington, USA

**Keywords:** biogeochemistry, ecological theory, ecosystem interfaces, historical contingency, hydrology, microbial communities, perturbation, resilience, scaling theory, succession

## Abstract

To improve predictions of ecosystem function in future environments, we need to integrate the ecological and environmental histories experienced by microbial communities with hydrobiogeochemistry across scales. A key issue is whether we can derive generalizable scaling relationships that describe this multiscale integration. There is a strong foundation for addressing these challenges. We have the ability to infer ecological history with null models and reveal impacts of environmental history through laboratory and field experimentation. Recent developments also provide opportunities to inform ecosystem models with targeted omics data. A major next step is coupling knowledge derived from such studies with multiscale modeling frameworks that are predictive under non-steady-state conditions. This is particularly true for systems spanning dynamic interfaces, which are often hot spots of hydrobiogeochemical function. We can advance predictive capabilities through a holistic perspective focused on the nexus of history, ecology, and hydrobiogeochemistry.

## Abstract (precis)

To improve predictions of ecosystem function in future environments, we need to integrate the ecological and environmental histories experienced by microbial communities with hydrobiogeochemistry across scales. A key issue is whether we can derive generalizable scaling relationships that describe this multiscale integration.

## PERSPECTIVE

**FIG 1:**
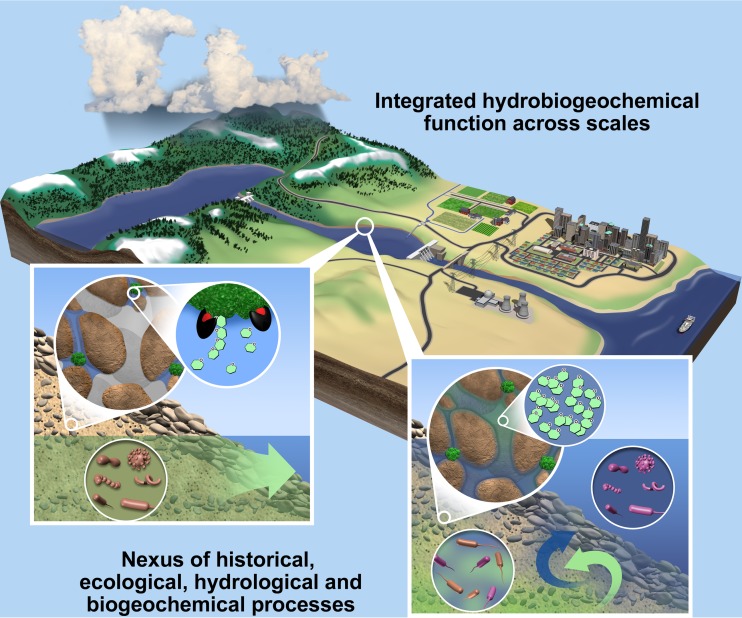
Conceptual overview of cross-scale connections among history, ecology, and hydrobiogeochemistry. The insets show a cross section of the subsurface below a stream (i.e., the hyporheic zone) under different hydrologic conditions, where green water and blue water represent groundwater and surface water, respectively. At the low-river stage (left inset), groundwater discharges and brings microbes from the aquifer (brown cells) into the hyporheic zone, resulting in an influence of dispersal. The low-river stage also causes pores to desaturate, which can spatially isolate organic carbon (green clumps) from microbial cells, and yet extracellular enzymes (black and red) continue to degrade particulate carbon into monomers that can accumulate. A rise in the river stage (right inset) causes groundwater-surface mixing, releases monomeric organic carbon, and increases the influence of selection. Ecologically similar taxa (same cell shapes) are thus selected for under mixed conditions, and biogeochemical function is elevated (not depicted). Hydrologic history therefore influences how systems respond ecologically and biogeochemically to a shift in hydrologic conditions. These interrelationships highlight the importance of the nexus among history, ecology, and hydrobiogeochemistry. A key issue is how processes at the scales shown in the insets impact hydrobiogeochemical function at the larger scales shown in the background. (Insets reproduced from reference 17.)

**FIG 2:**
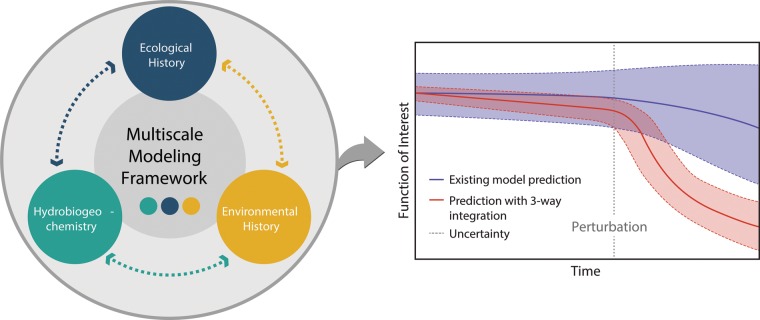
Summary of the conceptual hypothesis that accounting for history significantly shifts predictions of function. (Left panel) In natural systems, there is a dynamic three-way coupling among ecological history, environmental history, and hydrobiogeochemical function. Major challenges are those of understanding that coupling and, in turn, representing it within dynamic, non-steady-state multiscale modeling frameworks. (Right panel) Given clear evidence for a strong influence of history on function, it is expected that accounting for the three-way coupling will shift model predictions of function under future environmental conditions. It is expected that predictions of mean conditions (solid lines) will change, especially following perturbations, and that uncertainty will be reduced (solid filled areas). This formulation is conceptual, and the function is not specified but is considered to represent any microbe-influenced biogeochemical rate that is also impacted by hydrology.

The functioning of ecosystems—including the services that they provide to human society—is strongly governed by forms of feedback among hydrology, geochemistry, and the biological agents that drive biogeochemical transformations. This suite of integrated processes is conceptualized as “hydrobiogeochemistry,” and microbial communities are a central driver of ecosystem hydrobiogeochemical function (Fig. 1) (Table 1). It is becoming increasingly clear that the short-term (hours to days) and long-term (months to years) ecological and environmental history experienced by microbial communities must be integrated with hydrobiogeochemistry to improve predictions of ecosystem function (Fig. 2) (Table 1). In recent years, the scientific community has made significant progress toward this integration by combining omics (e.g., transcriptomics, metabolomics) and theory, and we are poised for additional breakthroughs. A major next step is using study outcomes linking history with hydrobiogeochemistry to inform multiscale predictive models.

**TABLE 1:** Glossary describing how terms are defined within this article, which may differ from definitions used elsewhere

Term	Definition
Assembly processes	Factors that govern the abundance of taxa within any given point in space or time; often split into deterministic and stochastic components
Deterministic selection	A collection of biotic and abiotic assembly processes that cause systematic differences in the success of different taxa within a given point in space or time; an example is low pH causing some taxa to be more successful than others
Dimensionless variables	Derived from the ratio of two variables that have the same units, whereby the resulting dimensionless numbers represent the relative magnitude of one variable over another; commonly used to characterize and predict system dynamics across scales
Dispersal limitation	A low rate of exchange of individuals between any two points in space; when dispersal limitation is combined with ecological drift, large differences in community composition between spatial locations can result
Earth system models	A class of simulation models that aim to model all major components of the integrated Earth system; often used to make spatially explicit predictions about future environmental conditions
Ecoevolutionary processes	The simultaneous operation of and feedback among factors influencing ecological (e.g., changes in relative abundances of taxa) and evolutionary (e.g., speciation) dynamics
Ecological drift	The ecological equivalent of “genetic drift” whereby unpredictable changes in the abundances of taxa occur due to random birth and death events; when dispersal between two locations is very low for multiple generations, ecological drift can cause large differences in community composition
Ecological succession	The progressive change in ecological conditions through time within a given spatial domain
Ecosystem function	Stocks and fluxes of energy and material within and through environmental systems; often conceptualized as providing services to human society, such as the transformation of pollutants (e.g., NO_3_^−^) by microbes into harmless forms
Homogeneous selection	Consistency through space or time in the factors that select for some taxa and against others; leads to the existence of communities that are ecologically similar through space or time
Homogenizing dispersal	A high rate of exchange of individuals between two points in space; results in similar community compositions across points in space
Hydrobiogeochemistry	The coupling among hydrology, geochemistry, and the biological agents (e.g., microbes, plants) that directly or indirectly influence biogeochemical transformations
Hydrobiogeochemical simulation models	Numerical models that represent coupled hydrobiogeochemical processes in order to understand and predict hydrologic, biogeochemical, and/or biological dynamics in environmental systems
Hydrologic inundation	Submersion of soils or sediments under a water column
Hyporheic zone	Spatial domain beneath and alongside running waters that is characterized by the mixing of surface water with groundwater
Metabolic scaling theory	A body of mathematical and conceptual constructs that link resource distribution networks within individuals to properties across scales (from individuals to ecosystems and from metabolic rate to long-term evolution)
Ecological null models	Randomization of ecological data to generate a pattern that is expected in the absence of specific ecological processes
Reaction network model	Mathematical model describing the progression of and coupling among biogeochemical reactions via a network structure; traditional models use a lumped description of biogeochemical pathways, e.g., including only initial reactants and final products, while advanced models account for detailed reactions and associated regulatory mechanisms
Source sink	A concept from population ecology in which one population of a given species can sustain itself and the dispersal of individuals out of that “source” population, arriving at and maintaining a second population (the sink) that would otherwise not persist due to unfavorable environmental conditions
Species richness	The number of unique species (or, more generally, of taxa) found within a given spatial domain or period of time
Stochasticity	Changes in the abundance of taxa within an ecological community that are not due to biotically or abiotically imposed systematic differences among taxa in the probability of successful reproduction
Variable selection	Heterogeneity through space or time in the factors that select for some taxa and against others; leads to ecologically divergent communities through space or time

Taking a mechanistic approach to predictive modeling that allows the outcome of finer-scale processes to be moved efficiently across scales will improve our ability to predict function under future environmental conditions. There is a particular need to move processes across scales using mechanistic simulation models that explicitly represent microbial communities, the reactions that they drive, and both the historical and contemporary hydrologic and biogeochemical processes that these communities interact with. This is especially true for systems that span dynamic interfaces such as the hyporheic zone (Table 1), tidally impacted systems, and others encompassing the terrestrial-aquatic continuum. For such systems, there are significant opportunities to develop predictive hydrobiogeochemical simulation models (Table 1) that couple physically based models of hydrologic transport—in both surface water and belowground water—with mechanistic biogeochemical reaction network models (Table 1) that explicitly represent microbial communities and their associated regulatory mechanisms. Some current models represent microbial communities explicitly (1), but these models do not account for history and have not been used within fully coupled hydrobiogeochemical models. It is also unclear how to bridge scales by leveraging fine-scale omics approaches to inform large-scale ecosystem predictions via microbially explicit models.

The influence of scale is pervasive (2), and it is a perennial challenge to understand how governing processes change with scale and how we can efficiently and effectively move knowledge of detailed mechanisms to larger scales within multiscale simulation models (3). For example, Stegen et al. (4) showed that the dominant drivers of soil respiration change with the spatial scale. It is, however, unclear how to use knowledge of that scale dependence to inform large-scale Earth system models (Table 1), which simulate all major components of the coupled Earth system in order to make predictions about future environmental conditions at the global scale. Metabolic scaling theory (Table 1) (5), however, has been very effective at linking fine-scale processes (e.g., resource distribution networks within individual organisms) to large-scale emergent phenomena (e.g., ecosystem-scale CO_2_ fluxes). For example, Stegen et al. (6) developed a simulation model based on fundamental ecoevolutionary processes (Table 1) informed by metabolic theory (e.g., the body size and temperature dependence of individual metabolic rate) to explain variations in large-scale gradients in species richness across a broad range of taxonomic groups (Table 1).

Existing work provides a foundation for linking history to hydrobiogeochemical function across scales. As discussed below, building from this foundation provides opportunities to achieve more-robust multiscale predictions of ecosystem function under future environmental conditions.

## ECOLOGICAL HISTORY IS REFLECTED IN COMMUNITY ASSEMBLY PROCESSES

Microbial community composition is the result of assembly processes (Table 1) that play out through time. Contemporary composition therefore reflects the history of assembly. Building from the work of Vellend (7), there has been significant progress made in quantitatively estimating relative influences of assembly processes. These estimates provide quantitative information about ecological history of a given study system and enable linkages between ecological history and hydrobiogeochemistry.

There are different ways of conceptualizing assembly processes, but quantitative estimates have focused on the relative influences of four categories referred to as variable selection, homogeneous selection, dispersal limitation combined with ecological drift, and homogenizing dispersal (Table 1) (8, 9). Variable selection occurs when selection pressures are distinct enough through space or time to deterministically (Table 1) drive large differences in the ecology of assembled individuals. Homogeneous selection occurs when selection pressures are similar enough through space or time to deterministically limit differences in the ecology of assembled individuals. Dispersal limitation results in the divergence of community composition—not necessarily the ecology of assembled individuals—when selection pressures are weak. In this case, random birth-death events lead to composition drifting apart through time (i.e., “ecological drift”). Homogenizing dispersal equates to mass effects, whereby high rates of dispersal cause similar compositions.

**FIG 3:**
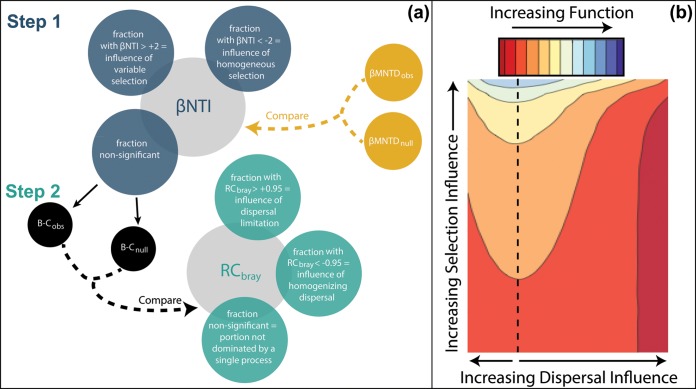
Null modeling approach for quantifying influences of assembly processes and connecting those processes to biogeochemical function. (a) In step 1, phylogenetic β-diversity is quantified with the beta-mean nearest taxon distance (β-MNTD) metric for all community pairs sampled within a given study system. The observed value (βMNTD_obs_) is compared to a null expectation when selection does not influence community assembly (βMNTD_null_). The deviation is quantified as the beta nearest taxon index (βNTI) metric, with significance thresholds of −2 and +2. The fractions of pairwise comparisons falling below or above those thresholds indicate influences of deterministic selection. In step 2, the pairwise comparisons that were nonsignificant are evaluated with a second null model that uses Bray-Curtis dissimilarity. Observed values (B-C_obs_) are compared to a null expectation when neither dispersal nor selection influences community assembly (B-C_null_). The deviation is quantified as the RC_bray_ metric, with significance thresholds of −0.95 and +0.95. The fractions of pairwise comparisons falling below or above those thresholds indicate influences of dispersal. (b) The contour plot (modified from reference 16) shows how increasing influences of selection lead to increased biogeochemical rates and that increasing influences of dispersal decrease biogeochemical rates. The vertical dashed line indicates the minimal influence of dispersal.

Stegen et al. (10) initiated an approach for estimating the relative influences of these four processes that can be applied to any set of communities for which information on community composition is available. The method uses a combination of two ecological null models (Table 1) that are based on comparing community compositions between pairs of communities contained within the larger set of communities (Fig. 3). One null model uses phylogenetic information to infer the influences of both types of selection, and a second null model is used to infer the relative contribution of each dispersal type. Resulting estimates have been used in a variety of ways to reveal linkages between history and hydrobiogeochemistry (11); these estimates can even reveal the presence and spatial structure of influential and yet unmeasured environmental variables (9).

## CONNECTING ECOLOGICAL HISTORY TO HYDROBIOGEOCHEMISTRY

Researchers have repeatedly found dispersal limitation and homogenizing dispersal to be important components of microbial community ecological history (9, 10, 12–14). This highlights the role of spatial processes and shows that microbial communities are not assembled purely by deterministic selection, in contrast to the Baas-Becking hypothesis, which suggests that “everything is everywhere, but the environment selects.” Signals of dispersal limitation indicate that the ecology of microbial communities can drift stochastically (Table 1) through time (15), suggesting that community members may not be locally optimized to drive biogeochemical reactions at maximal rates. In addition, homogenizing dispersal can rescue populations that would otherwise go locally extinct due to poor performance, as in the “source-sink” (Table 1) concept from population ecology. As such, both high and low rates of dispersal can lead to local microbial communities being composed of taxa/individuals that are minimally fit for their environment, leading perhaps to depressed biogeochemical function.

To connect assembly processes explicitly to biogeochemical function, Graham and Stegen (16) developed a simulation model in which assembly processes can be varied and resulting biogeochemical implications can be explored. Simulations showed that biogeochemical function decreases as the influence of deterministic selection decreases and as the influences of dispersal increase (Fig. 3). Across a range of systems, the relative balance between selection and dispersal varies systematically through space and time due to environmental drivers (9, 13, 14, 17) and successional dynamics (8, 18). Combining the theoretical outcomes from Graham and Stegen (16) with these observations indicates that there is a significant potential for spatiotemporal variation in the selection-dispersal balance to influence hydrobiogeochemical function. Mechanistic hydrobiogeochemical simulation models are needed that can account for this influence of ecological history, as well as the influence of environmental history.

## CONNECTING ENVIRONMENTAL HISTORY TO HYDROBIOGEOCHEMISTRY

To improve predictions of hydrobiogeochemical function under future environmental conditions, it is important to understand connections among environmental history, contemporary conditions, and responses to future change. Contemporary environmental conditions result from myriad processes playing out through time (i.e., environmental history) and represent the initial conditions that influence system responses to change.

Recent work provides intriguing outcomes that highlight the importance of environmental history for system response. For example, Bottos et al. (13) found landscape-scale spatial variation in a permafrost system under prethaw environmental conditions, reflecting the impact of topography on hydrologic history. A follow-on experiment showed that history strongly influenced microbial responses to thaw and depended specifically on the initial acetate concentration and organic carbon character (E. M. Bottos and J. C. Stegen, unpublished data). Goldman et al. (19) also revealed strong linkages between environmental history and hydrobiogeochemical function following perturbation. They found that a history of less-frequent hydrologic inundation (Table 1) within the hyporheic zone led to reduced biogeochemical function following reinundation. The researchers inferred that historical inundation dynamics governed the relative contributions of fungi and bacteria, in turn governing the biogeochemical response to reinundation.

**FIG 4:**
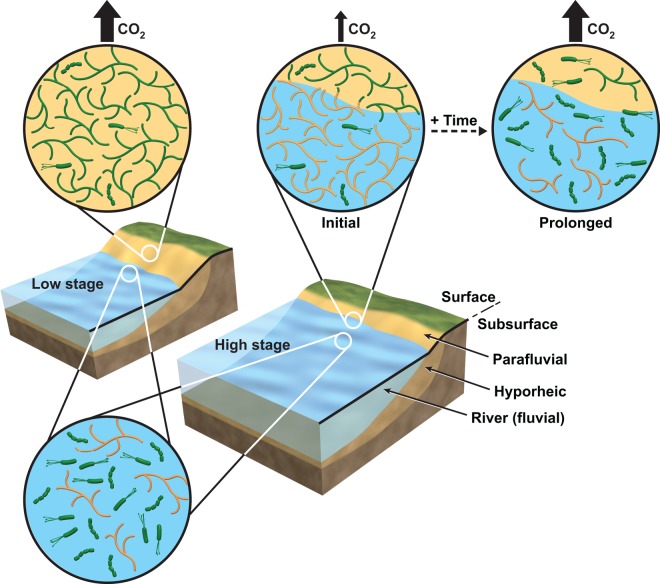
Conceptual model linking environmental history to biogeochemical response following perturbation. Goldman et al. (19) proposed that in infrequently inundated sediments (yellow background), fungi (branching organisms) perform well (green) but are metabolically suppressed (orange) following reinundation (blue shading). Given their dominance in the community, metabolic suppression leads to lower system-scale CO_2_ flux (thin arrow). Sustained inundation may shift the community toward bacterial (rod shapes) dominance, as observed in sediments that are more frequently inundated (lower left inset). With sustained inundation, high CO_2_ flux (thick arrow) may be regained due to reorganization of the microbial community. The time scale required for this recovery and the dependence of that time scale on historical conditions represent major knowledge gaps, however. (Modified from reference 19.)

Those two studies contribute to a number of other recent studies revealing how environmental history sets both biotic and abiotic initial conditions, strongly influencing the response to future perturbation (20). A significant knowledge gap, however, is that of how ecological and environmental histories influence the temporal scale of responses to perturbation. For example, while Goldman et al. (19) showed that environmental history influenced short-term (0.5-to-24-h) biogeochemical responses, the time scale to recover biogeochemical function is unknown (Fig. 4). Major challenges include characterizing these temporal dynamics, understanding governing processes and influences of history, and incorporating the resulting knowledge into predictive multiscale simulation frameworks.

## THE PATH AHEAD: INTEGRATING HISTORY WITH HYDROBIOGEOCHEMICAL FUNCTION ACROSS SCALES

A grand scientific challenge is that of using fundamental discoveries within microbial ecology to improve the ability of simulation models to predict system-scale hydrobiogeochemical function under future environmental conditions. One approach is that of integrating theory and empirical estimations of community assembly processes, concepts and influences of system history, and multiscale hydrobiogeochemical models. In doing so, we must consider that environmental systems are increasingly perturbed and dynamic. This is particularly true for systems spanning dynamic interfaces, which are often hot spots of hydrobiogeochemical function. It is therefore critical that that the simulation-based multiscale modeling frameworks used to integrate history with function should be able to handle very dynamic conditions across localized interfaces that strongly influence the hydrobiogeochemical function of the larger system.

As summarized above, there is a conceptual basis for integrating history with function, but we can go much further. In particular, ecological succession (Table 1) provides a framework that can encompass many key elements. Across stages of succession, there is significant temporal variation—ranging from 1 day to 100 years—in the balance between selection and dispersal (8, 18). Superimposing these observations with connections between the selection/dispersal balance and hydrobiogeochemical function suggests a three-way coupling among previous disturbance history, the temporal dynamics of assembly processes, and hydrobiogeochemical function. Major advances toward integrating history with function may be achieved by elucidating this three-way coupling through tight integration and feedback between modeling and experimentation. Furthermore, the three-way coupling among history, assembly, and function is relevant beyond hydrobiogeochemistry, having broad application across systems. For example, the gut microbiome’s influence on host health (i.e., system function) is modified by both history (e.g., preceding antibiotic use and subsequent resistance) and assembly processes (e.g., dispersal of pathogens into the gut). There is an opportunity to elucidate general principles by interrogating a broad range of systems using a consistent conceptual framework based on the history-assembly-function nexus.

We also need direct evaluation and modeling of how and why history and function (hydrobiogeochemical and beyond) influence each other across both temporal and spatial scales. There is great potential for progress by developing mechanistic models designed to use targeted omics data. For example, Song et al. (21) developed a regulation-based reaction network model that can be directly informed by transcriptomics and/or proteomics data. Using this model within field-scale hydrobiogeochemical simulation models provides an immediate opportunity to carry omics-derived insights to the ecosystem scale.

There are currently rapid and exciting advances at the nexus of ecology and hydrobiogeochemistry. Pursuing major outstanding questions will provide fundamental insights and enable improved predictions of function under future conditions. A key issue is whether we can derive generalizable scaling relationships that relate ecological and environmental history to hydrobiogeochemical function across scales. There is also a need to push beyond the concepts discussed above. For example, can we link history to function across scales by deriving dimensionless variables (Table 1)? Such variables may be based on the magnitude/duration of disturbances relative to historical conditions. A complementary approach to derive dimensionless variables could quantify organismal trait scaling (e.g., body size, dispersal ability) relative to the scaling of physical constraints imposed by porous media (i.e., soil and sediment) and hydrologic state. More generally, we will enable advanced predictive capabilities through a holistic perspective that predicts future function by accounting for the past and by leveraging data-model integration within dynamic multiscale modeling frameworks.
